# Reducing preterm mortality in eastern Uganda: the impact of introducing low-cost bubble CPAP on neonates <1500 g

**DOI:** 10.1186/s12887-019-1698-x

**Published:** 2019-09-04

**Authors:** F. Okello, E. Egiru, J. Ikiror, L. Acom, KSM Loe, P. Olupot-Olupot, K. Burgoine

**Affiliations:** 1 Mbale Campus, Busitema University, P.O.Box 1460, Mbale, Uganda; 2 Varimetrics Group Limited, P. O Box 2190, Mbale, Uganda; 3 Mbale Clinical Research Institute, P.O. Box 1966, Mbale, Uganda; 4 Neonatal Unit, Mbale Regional Referral Hospital, P.O. Box 1966, Mbale, Uganda; 5 Diamedica UK Ltd, Grange Hill Industrial Estate, Bratton Fleming, UK

**Keywords:** Bubble CPAP, Preterm, Respiratory distress syndrome, Neonate, Low-income country, Africa

## Abstract

**Background:**

Complications of prematurity are the leading cause of deaths in children under the age of five. The predominant reason for these preterm deaths is respiratory distress syndrome (RDS). In low-income countries (LICs) there are limited treatment options for RDS. Due to their simplicity and affordability, low-cost bubble continuous positive airway pressure (bCPAP) devices have been introduced in neonatal units in LICs to treat RDS. This study is the first observational study from a LIC to compare outcomes of very-low-birth-weight (VLBW) neonates in pre- and post-CPAP periods.

**Methods:**

This was a retrospective study of VLBW neonates (weight < 1500 g) in Mbale Regional Referral Hospital Neonatal Unit (MRRH-NNU), a government hospital in eastern Uganda. It aimed to measure the outcome of VLBW neonates in two distinct study periods: A 14-month period beginning at the opening of MRRH-NNU and covering the period until bCPAP was introduced (pre-bCPAP) and an 18-month period following the introduction of bCPAP (post-bCPAP). After the introduction of bCPAP, it was applied to preterm neonates with RDS when clinically indicated and if a device was available. Clinical features and outcomes of all neonates < 1500 g were compared before and after the introduction of bCPAP.

**Results:**

The admission records of 377 VLBW neonates < 1500 g were obtained. One hundred fifty-eight were admitted in the pre-bCPAP period and 219 in the post-bCPAP period. The mortality rate in the pre- bCPAP period was 39.2% (62/158) compared with 26.5% (58/219, *P* = 0.012) in the post-bCPAP period. Overall, there was a 44% reduction in mortality (OR 0.56, 95%CI 0.36–0.86, *P =* 0.01). There were no differences in birthweight, sex, presence of signs of respiratory distress or apnoea between the two groups.

**Conclusion:**

Specialized and resource-appropriate neonatal care, that appropriately addresses the challenges of healthcare provision in LICs, has the potential to reduce neonatal deaths. The use of a low-cost bCPAP to treat RDS in VLBW neonates resulted in a significant improvement in their survival in a neonatal unit in eastern Uganda. Since RDS is one of the leading causes of neonatal mortality, it is possible that this relatively simple and affordable intervention could have a huge impact on global neonatal mortality.

## Background

Complications of prematurity are the leading cause of deaths in children under the age of five [1]. In fact in 2015, complications from preterm birth led to the death of 1.055 million neonates worldwide, with the majority occurring in low- and middle-income countries (LMICs) like Uganda [1]. The predominant reason for these preterm deaths was RDS, with more than 50% of neonates born before 30 weeks of gestation developing RDS [2, 3]. In HICs, continuous positive airway pressure (CPAP) has been shown to reduce preterm mortality by 48%, reduce the need for mechanical ventilation by 50% and reduce chronic lung disease [4]. CPAP is now the standard of care for RDS in HICs, with the option of mechanical ventilation and artificial surfactant if required [5]. However, in LICs, ventilation, surfactant and CPAP are rarely accessible or affordable leaving limited treatment options for preterm neonates with RDS.

In Uganda, the Neonatal Mortality Rate has not changed over 2 decades, remaining high at 28/1000 live births and the leading cause of these deaths is complications of prematurity [6]. The UN Sustainable Development Goals seek to reduce global neonatal mortality to 12 deaths per 1000 live births by 2030 [7]. For this to be achieved, mortality from preterm complications must be drastically reduced. Dedicated and resource-appropriate neonatal care that appropriately addresses the challenges of healthcare provision in LICs is needed to help meet this goal. Low-cost bCPAP for preterm infants was listed by the World Health Organization in 2012 as an area in need of implementation [8].

Attempts at improvised CPAP are often made in LICs but this can be risky [9]. Due to their simplicity and affordability, low-cost bCPAP devices have been introduced in many neonatal units in LMICs [10]. However, there have been no randomized trials conducted in LMICs investigating the efficacy of bCPAP. Six observational studies from middle-income countries have compared outcomes of preterms in a pre- and post-CPAP periods [11–14]. In LICs, three observational studies in hospitals without access to mechanical ventilation or artificial surfactant, have documented the use of bCPAP [15–17].

## Aims

Our study is the first observational study in a LIC to determine the outcome of neonates < 1500 g during two distinct periods, before and after the use of bCPAP,

## Study location

The study was conducted in MRRH, a government hospital in eastern Uganda that serves a population of 4.5 million people. Since 11 May 2015, MRRH has had a dedicated NNU that admits over 2000 neonates a year including around 140 VLBW infants [18]. Neonates are admitted directly from the labour ward, referred from surrounding health facilities and, due to a high rate of home deliveries, some neonates are brought directly from home [6]. The MRRH-NNU is staffed by one full-time neonatal doctor, two neonatal clinicians, 6 specially trained neonatal nurses and a rotating intern. The ward has an average of 35 neonates admitted at any one time and 24-h nursing care is provided by a single neonatal specialist nurse in 8-hourly shifts.

## Standard of care

In MRRH-NNU, treatment with intravenous fluids, intravenous antibiotics, anticonvulsants, aminophylline, multivitamins and iron supplements is possible [18]. All VLBW infants are started on broad-spectrum antibiotics, aminophylline and dextrose 10% infusion on day 1. Almost all preterms are fed exclusively with expressed human breastmilk. Feeding is commenced at 25 ml/kg/day and advanced by 25 ml/kg/day as tolerated either by nasogastric tube or spoon feeding until 150 ml/kg/day is achieved. Thermoregulation is achieved using kangaroo care. MRRH-NNU does not routinely have access to echocardiography, blood gas analysis, c-reactive protein, blood cultures, blood pressure monitoring or portable chest x-ray. Mechanical ventilation and surfactant are not available.

Oxygen saturations are checked on admission and once daily on the ward round. Controlled free-flow nasal oxygen can be given from 0.1–1.5 l/min. On 12th July 2016, bCPAP was introduced in MRRH-NNU. The bCPAP devices (Diamedica UK Ltd., Bratton Fleming, UK) are designed for low-resource settings and can provide a distending pressure of up to 10cmH_2_O and a fraction of inspired oxygen (FiO_2_) between 0.21–0.95 [19]. The devices have an integrated oxygen concentrator, so cylinders are not required. Treatment was delivered using nasal prongs (RAM Cannula, Neotech) and a selection of sizes were available to ensure a suitable fit for each neonate. Laboratory testing by Diamedica showed that, if the water in the bubble bottle was at the correct level, the pressure setting on the bubble bottle was reflected at the RAM cannula (±1cmH_2_O) irrespective of the cannula size used.

Given the diagnostic limitations described above, RDS was a clinical diagnosis defined by signs of respiratory distress in a VLBW or preterm neonate. Indications to initiate bCPAP for RDS in MRRH-NNU included the presence of any of the following: severe subcostal recession, sternal recession, grunting, recurrent apnoea, hypoxia not responding to oxygen therapy. We were unable to exclude concurrent pneumonia or cardiac disease. At admission, all VLBW neonates are routinely given antibiotics for possible sepsis and aminophylline prophylaxis for apnoea of prematurity. The bCPAP was applied to any VLBW neonate with a clinical diagnosis of RDS if a device was available. Treating up to five neonates at one time. If a bCPAP device was not available the neonate continued on oxygen therapy alone as needed.

The bCPAP could be initiated by any member of staff including the nurses, therefore the MRRH-NNU bCPAP guideline was suitably simple. Every nurse working in the neonatal unit attended the Newborn Care Training Course, which included a 2-h module on the recognition and treatment of respiratory illness in neonates [18]. Each nurse was also given one-on-one bedside training from a senior member of the team in the recognition of respiratory distress, how to correctly set-up the bCPAP and how to select and apply the correct interface. The distending pressure was initiated at 5cmH_2_O and adjusted between 2-8cmH_2_O depending on the level of respiratory distress observed. The FiO_2_ was initiated at 0.58 and could be adjusted from 0.21–0.95 to achieve oxygen saturations of 90–93% in the preterm neonates [20].

bCPAP is a particular method of CPAP delivery that does not feature any sensors or closed loop feedback to control the pressure. Therefore, in any patient care using bCPAP, clinicians rely on clinical signs and titrate the pressure and the FiO_2_ depending on the needs of the patient. In this study, neonates on bCPAP settings were assessed 1–2 times a day by the neonatal doctor or neonatal clinician. The FiO_2_ was adjusted to maintain the oxygen saturations 90–93%. The pressure was adjusted between 2 and 8 cmH_2_O based on the level of respiratory distress evident on clinical examination. When no respiratory distress was present the pressure was reduced by 1–2 cmH_2_O and the clinical response observed. When respiratory distress and/or apneas were no longer present when using a pressure of 2 cmH_2_O, the neonate was weaned off bCPAP. This was done for increasing durations of time from 30 min up to 2 h daily. These periods were supported by oxygen therapy if required. Once the neonate was comfortable without bCPAP for a 2 h period it was discontinued.

## Methods

Two distinct study periods were identified in order to maximize the number of neonates inlcuded in the study. The first, (11 May 2015–11 July 2016) was a 14-month period representing the period from the opening of MRRH-NNU before the routine use of bCPAP machines (pre-bCPAP period). The second period (12th July 2016 – 31st Dec 2017) was the subsequent 18 months representing the period after bCPAP was introduced to MRRH-NNU (post-bCPAP period). The second period was limited by additional improvements in neonatal care in MRRH-NNU; the introduction of back-up power and continuous pulse-oximetry monitoring for all neonates on bCPAP in January 2018. The sample size reflects the limitations of the time periods described above.

Gestational age data was not routinely available at the time of this study and therefore birthweight was used as a proxy for prematurity. A birthweight < 1500 g is defined as VLBW [21]. VLBW corresponds approximately to < 32 weeks completed gestation, although high rates of growth restriction mean that prematurity may have been overestimated in this study. All VLBW neonates, with no severe congenital abnormalities were eligible for this study. The inpatient records for all VLBW neonates admitted to the NNU during the two study periods were identified. The following data elements were extracted: date of birth, date of admission, admission weight, gender, place of delivery, type of delivery. Need for resuscitation, defined as the need for ventilation ± chest compressions at delivery, was extracted from the referral document. Apgar scores were not often available on the referral document so were not included in the analysis. Vital signs at admission, signs of respiratory distress, presence of apnoea, method of respiratory support, length of hospital stay and in-patient outcome (discharge, died, referred) were also extracted.

Data from the pre-bCPAP period were compared with those admitted after the introduction of bCPAP. Data were analyzed using SPSS Version 25. Comparison of discrete variables between neonates before and after bCPAP was done using chi squared or fisher’s exact tests. For continuous variables, normal distribution was assessed using Shapiro Wilk Normality Test (Ho: Normal) yields *p* < 0.05). A student’s t-test was used to compare normally distributed continuous variables and a Mann-Whitney U test was used to compare medians. Two-sided *P*-values < 0.05 were considered statistically significant.

## Results

**Fig. 1:**
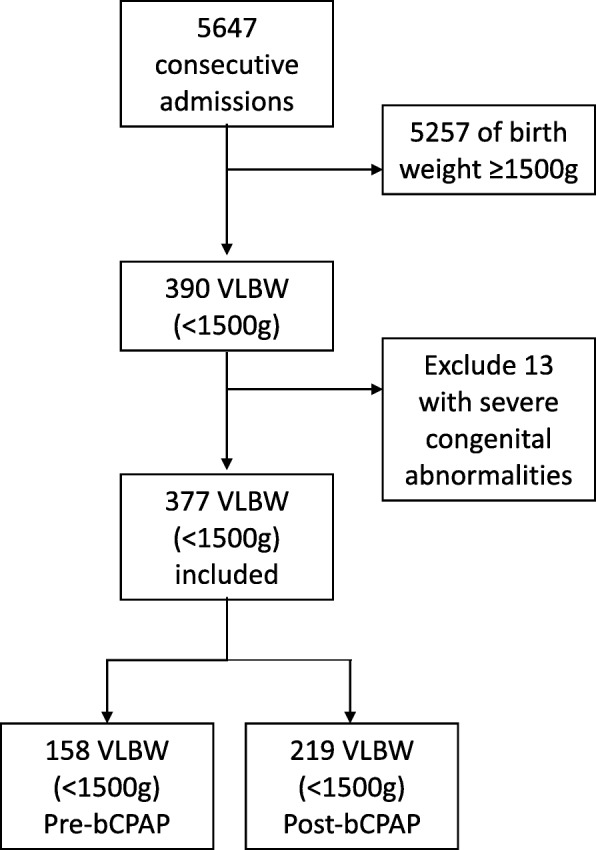
Flow chart of admitted and eligible neonates during the study period

During the study period 5647 neonates were admitted, of which 377 VLBW neonates were included. (Fig. 1) 158 were admitted in the pre-bCPAP period and 219 in the post-bCPAP period. During the post-bCPAP period, 25.6% (56/219) of VLBW neonates received bCPAP. The median duration of bCPAP for these neonates was 6 days (IQR 3, 10, range 1–62). There were no major statistical differences between the two groups in terms of place of birth, type of delivery or need for resuscitation at birth (Table 1). The admission weight, gender, admission observations and history of resuscitation were similar between the two groups. There was no difference in the admission vital observations between the groups, the presence of signs of severe respiratory distress or apnoea. There were significantly more neonates with subcostal recession in the post-bCPAP period suggesting this group of patients may have been sicker and therefore one might have anticipated worse outcomes.

**Table 1:** Demographics and outcomes of VLBW infants treated at the MRRH NNU

Characteristic	Pre-bCPAP period	Post-bCPAP period	*P* value, pre vs. post
Number of VLBW patients	158	219	–
Respiratory support			
- Oxygen only, N(%)	99 (62.7)	114 (52.1)	–
- bCPAP, N(%)	0 (0)	56 (25.6)	–
- Median (IQR) total days on oxygen and bCPAP (any respiratory support)	1 (0, 2)	2 (1, 4)	0.0000
Male sex (%)	64 (41.5)	107 (48.9)	0.25
Weight [g]			
- Median (IQR)	1230 (1070, 1370)	1210 (1050, 1346)	0.56
- Range	600–1490	480–1490	
- VLBW (1000-1499 g)	130/158 (82.3)	183/219 (83.6)	
- ELBW (< 1000 g)	28/158 (17.2)	36/219 (16.4)	
Mother’s age [years]			
- Median (IQR)	23.5 (20, 30)	22 (19, 27)	0.17
Place of delivery (%)			
- Hospital	89 (56.3)	126 (57.5)	
- Health Centre	36 (22.8)	69 (31.5)	
- Private clinic	2 (1.3)	9 (4.1)	
- Home	17 (10.8)	13 (5.9)	
- Other	9 (5.7)	0 (0)	
- Unknown	5	2 (0.9)	
Type of delivery (%)			
- SVD	129 (81.6)	189 (86.3)	
- Elective CS	1 (0.6)	0 (0)	
- Emergency CS	25 (15.8)	30 (13.7)	
- Unknown	3 (1.9)	0	
Resuscitation at birth			
- Yes (%)	51 (32.3)	91 (41.6)	0.13
Admission vitals			
- Temp [°C, mean ± SD)	35.2 ± 1.4	35.2 ± 1.2	0.98
- Heart rate [beats per min, median (IQR)]	139 (115, 152)	135.5 (119, 146)	0.31
- Oxygen saturations [%],median (IQR)]	89 (80, 93)	87 (76, 93)	0.21
- Subcostal recession	47/158 (29.8)	98/219 (44.8)	0.004
- Severe respiratory distress (sternal recession or grunting)	63/158 (39.9)	96/219 (43.8)	0.461
- Apnoea	16/158 (10.1)	24/219 (11.0)	0.866

Standard deviation (SD), Interquartile range (IQR)

The primary outcome, mortality rate (Table 2), was significantly lower in the post-bCPAP period (39.2% vs. 26.5%; *P =* 0.012). Overall, there was a 44% reduction in mortality (OR 0.56, 95%CI 0.36–0.86, *P =* 0.01). The reduction in mortality was similar in VLBW neonates (1000-1499 g) and extremely-low-birthweight (ELBW) neonates (< 1000 g), from 31.5 to 19.7% (*P =* 0.023) and 75 to 61.1% (*P =* 0.290) respectively. The impact in ELBW did not reach statistical significance; this was likely due to a relatively small number of ELBW neonates.

**Table 2:** Comparison of outcomes pre- and post-bCPAP

Characteristic	Period pre-bCPAP *n* = 158, N(%)	Period post-bCPAP *n* = 219, N(%)	*P* value, pre vs. post	Odds ratio pre vs. post (95% CI)
Outcome in hospital (%)				
- Discharged	79/158 (50.0)	128 (58.4)		
- Self-discharged	16/158 (10.0)	33 (15.0)		
- Died	62/158 (39.2)	58/219 (26.5)	0.012	0.56 (0.36–0.86)
- Unknown	1/158 (0.6)	0		
Hospital stay in days [median (IQR)]	8 (2, 17)	9.5 (4, 19)	0.26	
VLBW (1000-1499 g) mortality	31/130 (31.5)	36/183 (19.7)	0.023	0.53 (0.31–0.92)
ELBW (<1000 g) mortality	21 / 28 (75)	22/36 (61.1)	0.290	0.52 (0.14–1.74)

## Discussion

To our knowledge, this is the first retrospective study comparing a pre- and post-bCPAP era in a LIC. The survival of VLBW neonates at MRRH-NNU was significantly improved by the introduction of bCPAP for the treatment of RDS. This supports data from similar studies in MICs [11–14, 22, 23].

### Limitations

A critical analysis of this study’s limitations is vital. Firstly, we only evaluated a small number of VLBW neonates; this can be explained because we were a single center and were limited by the timings; the creation of the NNU, the introduction of bCPAP and further improvements to the NNU. Secondly, no accurate gestational data was available since none of the mothers had had a dating scan and the dates of the last normal menstrual period were often not known. The outcomes in this study can only be interpreted compared to birth weight. This may however provide a more pragmatic approach to clinicians working in similar low-resource settings, where even postnatal gestational age assessment can be challenging. It was not possible to ensure that all other variables remained constant during the two periods, however to our knowledge there were no changes in practice in MRRH labour ward during this time. In addition, no other equipment was introduced to the NNU apart from the bCPAP during the study. There were no changes in neonatal protocols, medications or training. Neonatal nursing staff in the NNU did change over during this period, although no additional staff were added. All neonatal nurses received the same neonatal training on commencing work in the NNU.

Resource limitations and the retrospective nature of the study means that there is limited data on adverse effects or complications of bCPAP. Future studies need to consider the incidence of nasal septum injury and pneumothorax. Our NNU chose to use binasal prongs to deliver bCPAP, due to their simplicity and availability of various sized prongs. It is possible that alternative interfaces may improve the delivery and the safety in similar settings.

It was not uncommon to have more neonates that required bCPAP than the five machines that were available. Unfortunately, data on these numbers were not available, therefore it is likely that the impact on mortality would be have been greater if more bCPAP machines were available. There is also a possibility that in practice staff tended to treat neonates with more severe RDS with bCPAP and those with less severe RDS with nasal oxygen. To account for this potential bias we compared the outcome of neonates who received bCPAP versus those who received only oxygen therapy in the post-bCPAP period. There was still a trend towards a lower mortality rate for those receiving bCPAP (13/56, 23.2%) than those neonates receiving oxygen alone (38/112, 33.9%, *P* = 0.22).

Failure of bCPAP can include death, pneumothorax, severe intraventricular hemorrhage and bronchopulmonary dysplasia. This study was not able to provide data regarding the risk factors, however previous studies have identified the FiO_2_, lower antenatal steroid exposure, birthweight, gestational age, apnoea, late initiation of CPAP, as significant predictors of CPAP failure [24–26]. These factors and indicators of failure need to be considered in future prospective studies of bCPAP.

### bCPAP in LMICs

Although there have been no randomized trials conducted in LMICs investigating the efficacy of bCPAP. Six observational studies from middle-income countries have compared outcomes of preterms in a pre- and post-CPAP periods [11–14]. Implementation of home-made bCPAP in India reduced mortality from 10.7 to 2.4% [11]. In another study in India, introduction of CPAP reduced up-transfers from 74 to 37% [13]. Findings were similar in South Africa, with up-transfers reduced from 16.7 to 5.1% [12]. In Kenya, the survival-to-discharge rate was increased from 61 to 85% after bubble CPAP was implemented [14]. A small retrospective study of VLBW neonates in Brazil showed the introduction of underwater bCPAP reduced the need for resuscitation, surfactant and mechanical ventilation in < 1500 g neonates [22]. In a large neonatal unit in Nicaragua, a new strategy to promote the systematic use of bCPAP significantly reduced the rate of intubation and mortality [23]. A small prospective observational study from India, that was able to monitor the safety of bCPAP with pulse oximetry, radiologically and with blood gases, found bCPAP to be safe [26].

In LICs, three observational studies in hospitals without access to mechanical ventilation or artificial surfactant, have documented the use of bCPAP [15–17]. In a dedicated physician-led neonatal ward in Malawi, implementation of a commercial low-cost bubble CPAP device for treatment of severe respiratory distress in neonates > 1000 g showed a 27% improvement in neonatal survival [17]. The study from Uganda, in a dedicated physician-led neonatal unit, and the study from Rwanda, in three rural district hospitals providing basic neonatal care, had no comparison groups and thus could only conclude that bCPAP was feasible for low-resource settings [15, 16]. The Rwandan study did observe a trend towards improved outcome of VLBW infants using bCPAP however they still reported low survival rates of VLBW infants treated with CPAP of 42%.

Despite the limitations of this study, it is clear that in a low-resource setting where blood gas analysis, x-rays, mechanical ventilation and surfactant administration are not routinetly available, bCPAP is an effective intervention for VLBW neonates with RDS. Not only is bCPAP low-cost, it also avoids the need for intubation and is simple to assemble and easy to apply. Given this simplicity, it is feasible to introduce bCPAP into a NNU that is mainly staffed by nurses and clinical officers, as was shown in South Africa [27]. This study suggests that with adequate training and supervision, low-cost bCPAP can have a dramatic impact on preterm mortality in such as setting. In fact, given the limited number of bCPAP machines, the reported reduction in mortality may actually be an underestimate.

Future research should focus on identifying which neonates benefit most from bCPAP and improving the data on the safety and complications of bCPAP. We must also establish the optimal time to initiate and discontinue bCPAP for these neonates and indeed whether prophylactic bCPAP in such settings with limited intensive care has benefit [26]. Data regarding the predictors of bCPAP failure would help identify those neonates that would benefit from intensive care if available. Lastly a minimum level of training and senior support for the safe and effective use of bCPAP needs to be established.

## Conclusion

The use of a low-cost bCPAP to treat RDS in VLBW neonates in a low-resource setting resulted in a significant improvement in their survival. More detailed and large-scale studies of the use, safety and indications for bCPAP in neonates in LICs are needed. It is possible that this relatively simple and affordable intervention could have a huge impact on global neonatal mortality.

## Data Availability

The datasets used and/or analysed during the current study are available from the corresponding author on reasonable request.

## Abbreviations

bCPAP: Bubble continuous positive airways pressure
CPAP: Continuous positive airways pressure
ELBW: Extremely-low-birthweight
HIC: High-income country
LIC: Low-income country
LMIC: Low- and middle-income countries
MRRH: Mbale Regional Referral Hospital
NNU: Neonatal Unit
RDS: Respiratory distress syndrome
VLBW: Very low-birthweight
